# Identification of Corosolic and Oleanolic Acids as Molecules Antagonizing the Human RORγT Nuclear Receptor Using the Calculated Fingerprints of the Molecular Similarity

**DOI:** 10.3390/ijms23031906

**Published:** 2022-02-08

**Authors:** Joanna Pastwińska, Kaja Karaś, Anna Sałkowska, Iwona Karwaciak, Katarzyna Chałaśkiewicz, Błażej A. Wojtczak, Rafał A. Bachorz, Marcin Ratajewski

**Affiliations:** 1 Laboratory of Epigenetics, Institute of Medical Biology, Polish Academy of Sciences, 93-232 Lodz, Poland; jpastwinska@cbm.pan.pl (J.P.); kkaras@cbm.pan.pl (K.K.); asalkowska@cbm.pan.pl (A.S.); isachrajda@cbm.pan.pl (I.K.); katarzyna.chalaskiewicz@onet.pl (K.C.); 2 Centre of New Technologies, University of Warsaw, 02-097 Warsaw, Poland; blazej.wojtczak@gmail.com; 3 Laboratory of Molecular Modeling, Institute of Medical Biology, Polish Academy of Sciences, 93-232 Lodz, Poland; rafal@bachorz.eu

**Keywords:** RORgammaT, RORC, Th17, inverse agonist, corosolic acid, oleanolic acid, calculated fingerprints of the molecular similarity, tanimoto similarity

## Abstract

RORγT is a protein product of the RORC gene belonging to the nuclear receptor subfamily of retinoic-acid-receptor-related orphan receptors (RORs). RORγT is preferentially expressed in Th17 lymphocytes and drives their differentiation from naive CD4+ cells and is involved in the regulation of the expression of numerous Th17-specific cytokines, such as IL-17. Because Th17 cells are implicated in the pathology of autoimmune diseases (e.g., psoriasis, inflammatory bowel disease, multiple sclerosis), RORγT, whose activity is regulated by ligands, has been recognized as a drug target in potential therapies against these diseases. The identification of such ligands is time-consuming and usually requires the screening of chemical libraries. Herein, using a Tanimoto similarity search, we found corosolic acid and other pentacyclic tritepenes in the library we previously screened as compounds highly similar to the RORγT inverse agonist ursolic acid. Furthermore, using gene reporter assays and Th17 lymphocytes, we distinguished compounds that exert stronger biological effects (ursolic, corosolic, and oleanolic acid) from those that are ineffective (asiatic and maslinic acids), providing evidence that such combinatorial methodology (in silico and experimental) might help wet screenings to achieve more accurate results, eliminating false negatives.

## 1. Introduction

Th17 lymphocytes are one of the subsets of T-helper cells that secrete proinflammatory cytokines, including IL-17A, IL17F, IL-21, IL-22, and GM-CSF2. These lymphocytes contribute to pathogen (e.g., *Bacillus anthracis* [1], *Candida albicans* [2], *Staphylococcus aureus* [3]) clearance from mucosal barriers and play a role in their maintenance [4,5]. However, the overactivation of Th17 cells is associated with the pathogenesis of certain autoimmunological diseases, such as psoriasis [6], psoriatic arthritis [7], rheumatoid arthritis [8], multiple sclerosis [9], Crohn’s disease [10], and ankylosing spondylitis [11].

RORγT is considered a master regulator of Th17 differentiation [12]. This transcription factor belongs to the nuclear receptor subfamily of retinoic-acid-receptor-related orphan receptors (RORs), which also includes RORα (NR1F1) and RORβ (NR1F2) [13,14], and is one of two isoforms of the RORC gene (NR1F3). The second protein product of the RORC gene is RORγ, which is 21 amino acids longer than RORγT. RORγ, in contrast to RORγT, is expressed in many tissues [15]. The expression of RORγT is restricted to the set of some immune cells, being the highest in Th17 lymphocytes [12,16,17]. RORγT, similar to the other nuclear receptors [18], has a ligand-binding domain and modulates gene activation/repression by binding coactivators or corepressors in a ligand-dependent manner [19,20]. Because RORγT is involved in the development of the immune system and is also associated with the pathogenesis of some autoimmune diseases and because its activity is regulated by small molecules, it is a putative target for drug design [21]. Among the compounds that interact with the RORγT ligand-binding protein are agonists that activate the receptor [22] and inverse agonists that inhibit the activity of the receptor [23]. The latter molecules are particularly interesting because they may be used to treat patients suffering from autoimmune diseases.

In a world where we have an ever-increasing number of compounds, the efficiency and accuracies of standard screening methods seem insufficient [24] for searching chemical libraries to find new drug candidates. Therefore, increasingly evolving computer methods aid the process of either screening or rational drug design [25]. In this work, we reanalyzed a previously experimentally screened chemical library [26] using the calculated fingerprints of molecular similarity, and we found compounds that are very similar to the RORγT inverse agonist ursolic acid [27]. Further investigations using a cellular reporter system and analysis of the expression of Th17-specific genes/cytokines in Th17 lymphocytes allowed us to identify compounds exerting stronger biological effects than those that were less effective.

## 2. Results

### 2.1. Computational Identification of Novel RORγT Inverse Agonists

**Figure 1:**
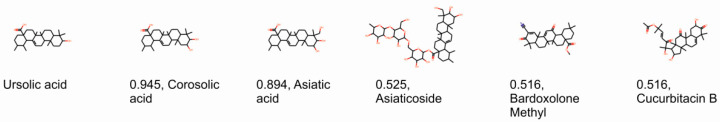
Identification of corosolic and asatic acids as compounds similar to ursolic acid in a virtual screening of the L1600 Kinase Inhibitor Library (TargetMol).

**Figure 2:**
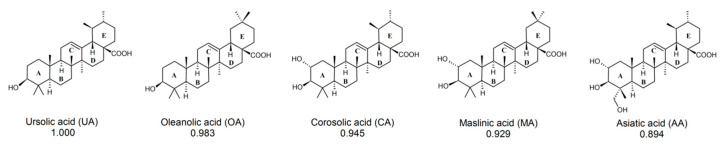
Tanimoto similarity values of the ursolic acid analogs.

**Figure 3:**
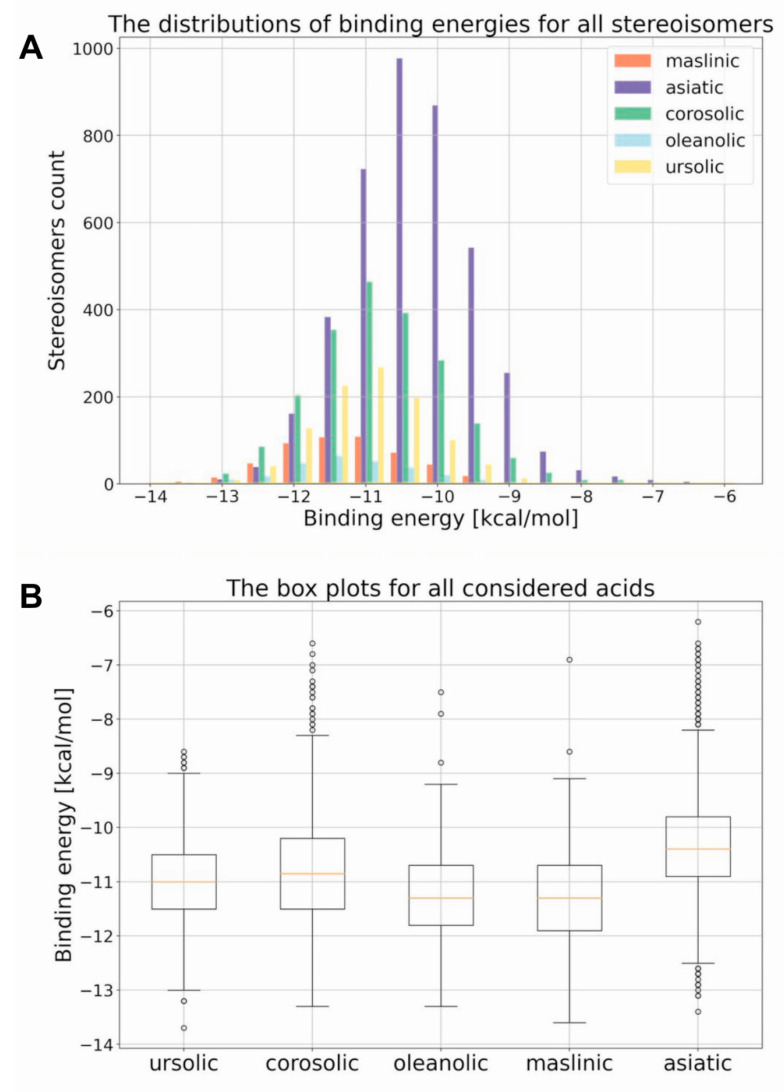
Molecular docking analysis of the ursolic, corosolic, oleanolic, asiatic, and maslinic acids binding to the LBD of the RORγ receptor. (**A**) Histograms of the binding energies of all considered acids with the 3l0j receptor. (**B**) Boxplots of the binding energies of all considered acids with the 3l0j receptor.

Previous research (including our own) identified ligands for RORγ/RORγT and other nuclear receptors based on screenings of chemical libraries. This involved a labor-intensive, costly, and time-consuming period of searching for a compound possessing the desired characteristics. Thus, we decided to use powerful chemoinformatic tools for the virtual screening of previously experimentally analyzed chemical libraries (L1600 Kinase Inhibitor Library, TargetMol) [26]. For the entire library, topological 2048-bit fingerprints were calculated (the 2048-element bit vector reflecting the topological properties of the molecule). Then, 11 molecules were selected (based on the available literature) as reference well-binding and active RORγT ligands: digoxin, 20,22-dihydrodigoxin, beta-acetyldigoxin (Oprea1_343674), cholesterol, 25-hydroxycholesterol, 7 alpha-hydroxycholesterol, cholesterol sulfate, ursolic acid, SR1001, SR1555, and T0901317 [27,28,29,30,31,32,33,34,35]. Based on the calculated fingerprints, the molecular similarity (Tanimoto similarity) was estimated for each pair: experimentally verified ligand–ligand from the library. Exceptionally high similarity was found for ursolic acid (previously identified RORγT inverse agonist [27]); the two most similar species, i.e., corosolic acid and asiatic acid, have Tanimoto scores of 0.945 and 0.894, respectively (Figure 1). Because these compounds belong to the larger group of pentacyclic triterpenoids, based on a literature search, we decided to include other ursolic acid analogs in the analysis: oleanolic and maslinic acids [36,37,38]. The similarity of oleanolic acid to ursolic acid and maslinic acid to ursolic acid was 0.983 and 0.929, respectively (Figure 2). To obtain a better understanding of the biological potential of the considered species, we carried out molecular docking calculations. All five acids have a number of chiral centers, and knowledge about absolute configuration is unfortunately not available. Therefore, we decided to carefully consider the entire space of stereoisomers. In Table 1, we present the number of chiral centers and the resulting number of stereoisomers for each considered acid. Thus, we encountered the same problem previously identified by Brink and Exner; we do not know if the steroisomer present in the database or generated using computational methodology is active toward the considered receptor [39]. The optimal pose search space was determined by a cube with an edge length of 25 Å centered in the geometrical center of the 3l0j native ligand. The explicit treatment of all stereoisomers is an approach that thoroughly searches the stereochemical space, but due to the significant number of stereoisomers (256, 512, 1024, 2048, and 4096 for maslinic, oleanolic, ursolic, corosolic and asiatic acids, respectively), the discussion about the results becomes more complex. Since we did not have any presumptions about the stereochemistry, we did not focus on a particular stereoisomer. Instead, we present the complete distributions of binding energies between the considered acids and the RORγ receptor. Figure 3A presents the histograms of the resulting binding energies between all considered acids and the 3l0j receptor. For each considered compound, we found a different number of stereoisomers, and the height of the bars varied. The distributions of binding energies form a Gaussian-like shape. In the case of asiatic acid, the distribution was shifted to the right, which was also reflected in the experiment (see next section). For the remaining species, we did not see significant differences. Figure 3B shows the box plots of all the distributions. The minimum, maximum, and median values were as follows: ursolic acid: −13.7 kcal/mol, −8.6 kcal/mol, and −11.0 kcal/mol; corosolic acid: −13.3 kcal/mol, −6.6 kcal/mol, and −10.85 kcal/mol; oleanolic acid: −13.3 kcal/mol, −7.5 kcal/mol, and −11.3 kcal/mol; and maslinic acid: −13.6 kcal/mol, −6.9 kcal/mol, and −11.3 kcal/mol. In the case of asiatic acid, both the median (−10.4 kcal/mol) and maximum values (−6.2 kcal/mol) of the binding energy were slightly higher than those of the remaining species (Table 2).

**Figure 4:**
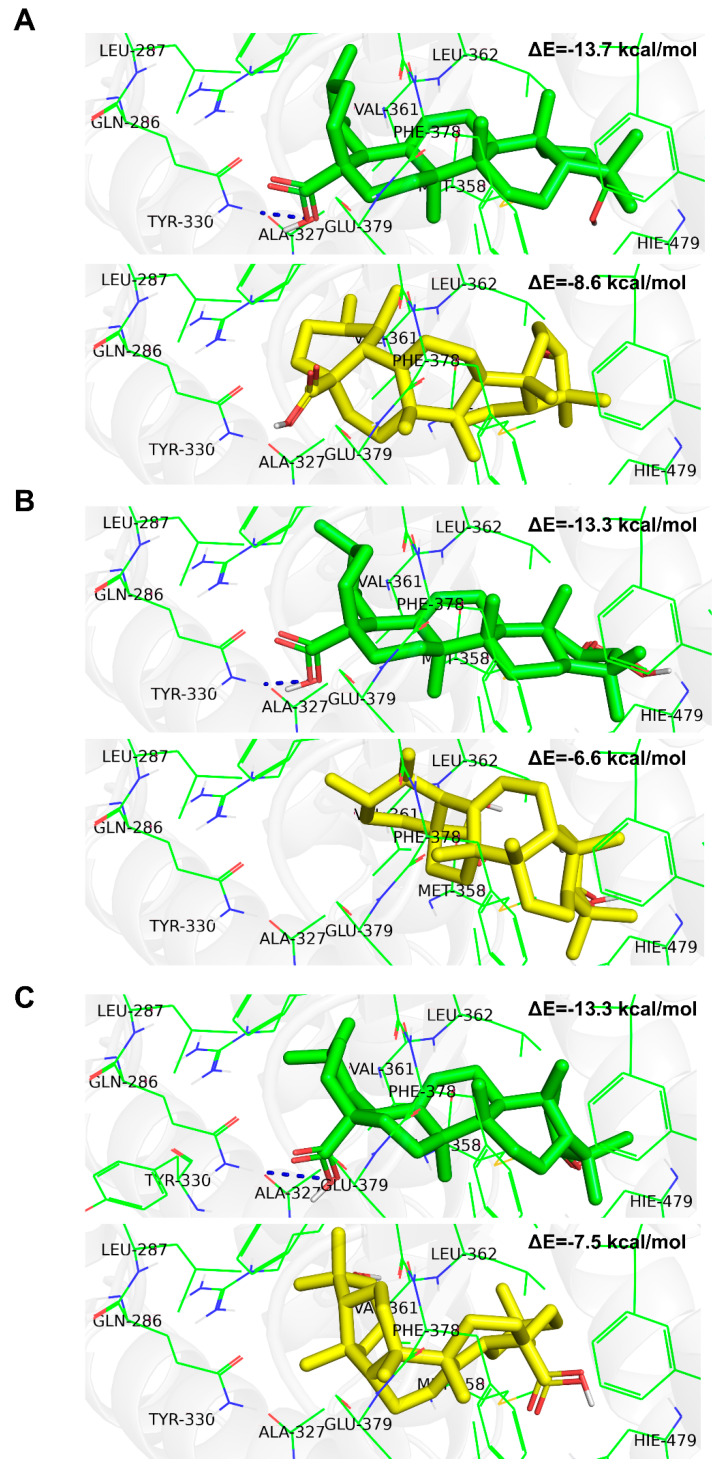
Molecular docking analysis of the best (green) and the worst (yellow) stereoizomers for ursolic (**A**), corosolic (**B**), and oleanolic (**C**) acids binding to the LBD of the RORγ receptor. Hydrogen bonds are represented as dark-blue dotted lines.

In order to provide a basic understanding of how the stereochemistry impacts the protein–ligand interaction we have prepared the visualizations of the best and worst stereoisomer, considered from the perspective of binding affinity. All considered acids are composed of five non-aromatic rings substituted with a varying number of carboxyl/hydroxyl groups, which should be considered as hydrogen bond donors or acceptors. Due to the lack of aromatic rings we did not expect any π-π or dispersion interactions; the entire binding should be related to the polar interactions between relevant groups. This is certainly the case. For instance, in the case of ursolic acid, one can clearly see that the stereoisomer characterized with the best binding affinity (Figure 4A) formed a hydrogen bond between carboxyl group and amino group of the GLN-286 residue. On the other hand, this was not the case of the stereoisomer, which reflected the weakest interaction with the protein (Figure 4A). Analogous behavior was seen for the remaining species (Figure 4B,C and Figure S2). Maslinic acid is an extreme example. The best stereoisomer occupies the receptor’s binding pocket and forms a strong interaction with the GLN-286 amino acid, which results in a low binding energy (Figure S2). The geometry of the worst stereoisomer, on the other hand, is unfavorable, and the docking procedure led to a position outside of the binding pocket (Figure S2). This obviously results in overall less intensive protein–ligand interactions, which makes this particular stereoisomer the worse drug candidate by far.

### 2.2. Experimental Confirmation of Inverse Agonistic Properties of Corosolic and Oleanolic Acids

**Figure 5:**
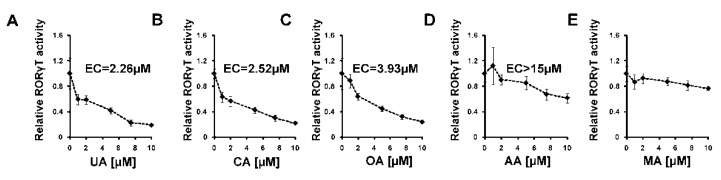
Effect of ursolic acid analogs on RORγ-dependent transcription in the HEK293 cell line. HEK293 cells were cotransfected with the pGL4.35[luc2P/9XGAL4UAS/Hygro], GAL4-DBD RORγ, and pCMVSEAP vectors. Twenty-four hours later, the cells were treated with increasing concentrations of ursolic (**A**), corosolic (**B**), oleanolic (**C**), asiatic (**D**), and maslinic acids (**E**) for another 48 h. After that time, the cells were lysed, and luciferase activity was measured. Luciferase results are standardized for the transfection efficiency control, which was SEAP. Mean ± SD, *n* = 3. EC50 values were calculated using AAT Bioquest (Sunnyvale, CA, USA) EC50 calculator, (https://www.aatbio.com/tools/ec50-calculator/, accessed on 6 December 2021).

**Figure 6:**
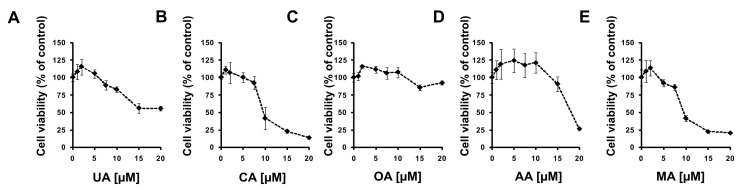
(**A**–**E**) Effect of ursolic acid analogs on CD4+ lymphocyte viability. CD4+ cells were isolated from buffy coats of healthy donors and subjected to Th17 polarization in the presence of increasing concentrations of ursolic, corosolic, oleanolic, asiatic, and maslinic acids for 5 days. Then, cell viability was determined using the CellTiter-Glo^®^ Luminescent Cell Viability Assay. Mean ± SD, *n* = 3, compared with control cells.

**Figure 7:**
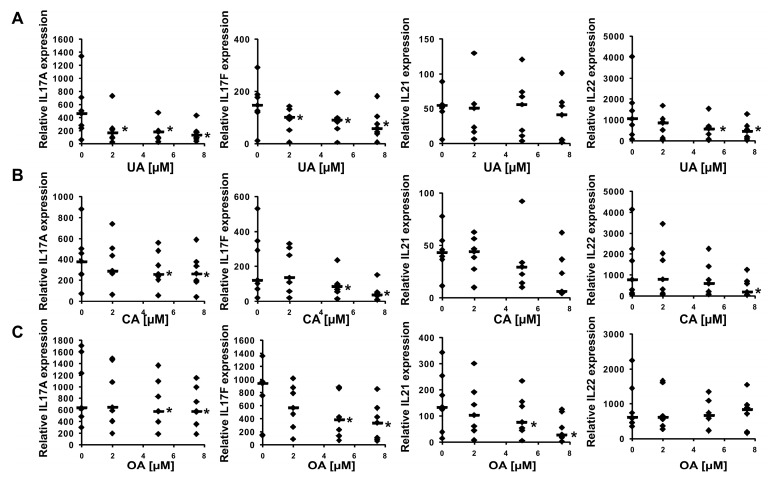
Effect of ursolic acid analogs on the expression of selected genes in human Th17 cells. Human naive CD4+ cells were treated with increasing concentrations of ursolic (**A**), corosolic (**B**), and oleanolic (**C**) acids and cultured under Th17-polarizing conditions for 5 days. Then, cells were collected for RNA extraction. The expression of the *IL17A*, *IL17F*, *IL21*, and *IL22* genes was determined by real-time RT–PCR. The results were normalized to the housekeeping genes *HPRT1*, *HMBS*, and *RPL13A*. An asterisk indicates a statistically significant difference at *p* < 0.05 compared with control cells. The data are presented as statistical dot plots with the median value (bars) from seven independent cultures (*n* = 7).

**Figure 8:**
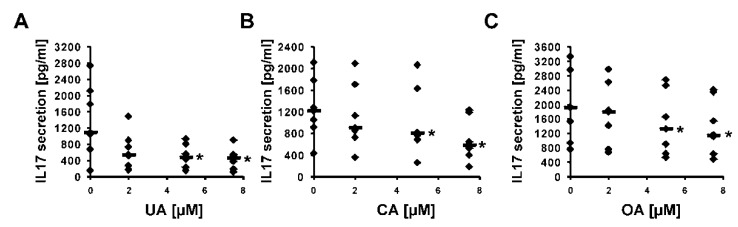
The analysis of IL-17 production in supernatants of Th17 cells cultured in the presence of increasing concentrations of ursolic (**A**), corosolic (**B**), and oleanolic acids (**C**) for 5 days was determined using the Quantikine Human IL-17 Immunoassay kit (R&D Systems). An asterisk indicates a statistically significant difference at *p* < 0.05 compared with control cells. The data are presented as statistical dot plots with the median value (bars) from seven independent cultures (*n* = 7).

**Figure 9:**
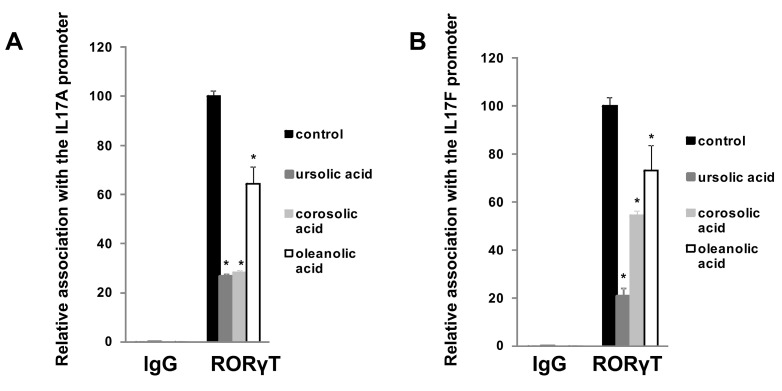
Chromatin immunoprecipitation results showing that ursolic, corosolic, and oleanolic acids decrease the levels of RORγT protein occupancy on the *IL17A* (**A**) and *IL17F* (**B**) gene promoters. Mean ± SD, *n* = 3, an asterisk indicates a statistically significant difference at *p* < 0.05 compared with control.

First, we decided to check how these five considered compounds affect RORγ transactivational activity in a cellular reporter system. We used the GAL-RORγ-LBD reporter described previously [35] to check whether these compounds could inhibit the constitutive activity of RORγ. Gene reporter assays showed that ursolic, corosolic, oleanolic, and, to some extent, asiatic acids, were able to decrease the activity of the RORγ reporter (Figure 5A–D) in HEK293 cells in a dose-dependent manner, while maslinic acid did not show such properties (Figure 5E). We then explored the effects of these compounds on RORγT-dependent expression in Th17 cells. First, we checked the cytotoxicity of these compounds toward Th17 cells. As shown in Figure 6, we did not observe the cytotoxicity of the analyzed acids up to 7.5 μM. We then differentiated CD4+ cells isolated from the buffy coats of healthy donors into Th17 lymphocytes in the presence of ursolic, corosolic, oleanolic, asiatic, and maslinic acids for 5 days, and then the expression of *RORγT*, *IL17A*, *IL17F*, *IL21*, *IL22*, and *APOD* was analyzed. Ursolic, corosolic, and oleanolic acids led to a decrease in the expression of *IL17A* (by 71%, 41%, and 10%, respectively, for the highest concentration that was used) and *IL17F* (by 60%, 70%, and 66%, respectively, for the highest concentration that was used) (Figure 7). Asiatic acid did not influence the expression of *IL17A*, and it inhibited the expression of *IL17F* (by 47%) at the highest concentration that was used (Figure S3). Maslinic acid was ineffective toward both (*IL17A/F*) genes (Figure S3). None of the compounds affected the expression of *RORγT* and *APOD* (Figure S4). Interestingly, in cells treated with ursolic and corosolic acids, substantially decreased expression of *IL22* was observed (by 66% and 75%, respectively, for the highest concentration that was used), and the expression of *IL21* was unaffected (Figure 7), while in cells treated with oleanolic and asiatic acids, we did not observe changes in the expression of *IL22*, but the expression of *IL21* was impaired (by 79% and 45%, respectively, for the highest concentration that was used) (Figure 7 and Figure S3). ELISA also confirmed that IL-17 secretion was diminished in the supernatants of the cells cultured in the presence of ursolic, corosolic, and oleanolic acids (Figure 8), with the strongest inhibition observed for ursolic and corosolic acids (by 57% and 52%, respectively). We did not observe any effects of the treatments of Th17 cells with asiatic and maslinic acids (Figure S5), which suggests that both acids show very weak or do not show any inverse agonistic properties toward RORγT. To determine whether all three positive compounds are able to decrease the binding of the RORγT transcription factor from the promoters of its target genes, chromatin immunoprecipitation was performed in Th17 cells. In control cells, *IL17A* and *IL17F* promoters were highly occupied by the RORγT protein; however, treatment with ursolic, corosolic, and oleanolic acids decreased the binding of this transcription factor and confirmed that these compounds act in an inverse agonistic manner (Figure 9). Again, it should be noted that ursollic and corosolic acids showed stronger inhibitory effects than oleanolic acid.

## 3. Discussion

The role of the RORγT receptor in the development of Th17 lymphocytes and the possibility of modulating its activity by the use of specific ligands make it an attractive target for the search for substances that may find application in the treatment of autoimmune diseases. Clinical studies with antibodies neutralizing interleukin 17 (e.g., ixekizumab, bimekizumab [40,41]) indicate that the use of the inverse agonists of RORγT that regulate the expression of IL17 [16] may also be effective or may increase the effectiveness of these antibody actions in combination therapy. To date, 17 compounds have entered clinical trials [42], and new drug candidates are being discovered. However, some of these trials have been halted due to the lack of satisfactory results, toxicity, and tumor induction [43]; thus, new compounds with more effective action are needed.

In the present study, using cheminformatic tools, we reanalyzed a previously experimentally screened library and identified corosolic acid and asiatic acid as compounds with a high similarity to ursolic acid. A further literature search indicated that oleanolic and maslinic acids have structures with high similarity to ursolic acid [38]. All isomeric structures are based on pentacyclic triterpenoid structures, which consist of five six-membered rings A-E. The simplest structure belongs to ursolic acid, and although all structures have similar formulas, they differ in the number of methylene (CH3) and hydroxy (OH) groups. All compounds possess one hydroxy group at the C3 position of the six-membered ring A and a double bond at the C12 position of ring C. However, corosolic acid at the C2 position has one additional hydroxy group in the trans conformation relative to the first hydroxy group. Asiatic acid has, in addition to these two groups, a third hydroxyl group located at one of the methylene groups exactly at the C23 position. There are few differences between oleanolic and maslinic acids compared to ursolic acid. The most significant difference concerns the E ring, which lacks a methylene group at the C19 position but contains an additional methylene group at the C20 position. The remaining oleanolic or maslinic acid structures (rings A–D) are similar to ursolic and corosolic acids, respectively. Interestingly, when we analyzed the in silico binding of all these compounds to the ligand-binding domain of RORγT, we did not find significant differences among them (Figure 3). An interesting and quite often skipped point in the literature is that the binding energy for different stereoisomers of the same compound changes significantly, and this also applies to other parameters such as protonation [44]. Many homochiral compounds have certain desirable biological properties (eutamers) in contrast to others that do not exhibit such properties or show opposite biological effects (distomers). Therefore, in drug development, more and more attention is paid to make new compounds homochiral, especially since the affinity of a given stereoisomer to a biological receptor is usually different [45,46]. This was also seen in the analysis we performed where we observed significant differences in binding energies between stereoisomers, which are also dependent on the number of chiral centers in the compound. As shown in Figure 4 and Figure S2, the stereochemistry of the species plays a significant role in the intensity of the protein–ligand interaction. In the case of the considered acids the decisive factor is the ability of particular stereoisomer to form the hydrogen bond with the GLN-286 amino acid. The stereochemistry to a large extent determines the conformation of the six-member rings and affects the overall shape of the molecule. In all the worst cases the species are bent, but the extent of this behavior depends on the acid. The maslinic acid is an extreme example. The best stereoisomer occupies the receptor’s binding pocket and has a strong interaction with the GLN-286 amino acid, which results in a low binding energy of −13.6 kcal/mol (Figure S2B). The geometry of the worst stereoisomer, on the other hand, was severely bent, and docking procedure led to a position outside of the binding pocket (Figure S2B). This obviously resulted in overall less intensive protein–ligand interactions characterized by a binding energy of −6.9 kcal/mol, which makes this particular stereoisomer a significantly worse drug candidate. The impact of stereochemistry on the shape of the molecule was also clearly visible in the oleanolic and asiatic acids cases (Figure 4C and Figure S2A). In all the considered cases this influence can be quantified by comparing the minimum and maximum binding energy (Table 2).

Experimental data using the RORγ-LBD-GAL4 system confirmed that ursolic (EC = 2.26 μM), corosolic (EC = 2.52 μM), oleanolic (EC = 3.93 μM), and, to some extent, asiatic (EC > 15 μM) acids exert inverse agonistic properties toward RORγT (Figure 5, Figure 7, Figure 8 and Figure 9), while maslinic acid does not. It is interesting that compounds possessing almost identical structures differ so much in their biological activities. However, Yukawa et al. showed [47] that even a small change in the structure of the compound interacting with the RORγT LBD can lead to a profound change in its properties toward this receptor. Analysis of the cell viability of CD4+ lymphocytes differentiated into Th17 cells in the presence of increasing concentrations of these compounds revealed that they were not cytotoxic up to 7.5 µM (Figure 6). CD4+ cells differentiated into Th17 lymphocytes in the presence of increasing concentrations of ursolic, corosolic, and oleanolic acids showed the diminished expression of *IL17A/F* and IL17 secretion (Figure 7 and Figure 8). Interestingly, ursolic and corosolic acids led to a decrease in the expression of *IL21*, while oleanolic and asiatic acids decreased the expression of *IL22* (Figure 7), suggesting that the mechanism of action or the choice of target gene might vary slightly depending on the compound. This difference might be explained by the off-target proteins interacting with each of the analyzed compounds or by their ability to recruit different coactivators or corepresors and/or binding to different response elements in the regulatory sites as was evidenced previously for PXR ligands, e.g., bisphenol A, phthalate, or pregnenolone, which induced PXR-dependent *CYP3A4* but not *ABCB1*. Interestingly, cisplatin induced much more expression of *ABCB1* in comparison to *CYP3A4* [48,49]. Chromatin immunoprecipitation also confirmed that ursolic, corosolic, and oleanolic acids decrease the binding of RORγT from the promoters of the *IL17A* and *IL17F* genes (Figure 9).

We identified herein that corosolic and oleanolic acids have similar properties to ursolic acid. All these compounds are pentacyclic triterpenoids that are found in several plants, including herbs, spices, and fruits [50]. It was previously demonstrated that pentacyclic triterpenoids isolated from loquat leaves inhibited rodent Th17 cells, alleviated renal pathological damage, and reduced skin inflammation in a mouse model [51,52]. Interestingly, some of the considered compounds have anti-inflammatory properties, e.g., corosolic acid inhibits the LPS-mediated activation of IRAK-1 and acute inflammation [53]; ursolic acid targets CASP3, ERK, and JNK2 and their effector transcription factors, and alleviates inflammation [54]. Clinical trials with oleanolic and ursolic acids [55,56,57,58,59] revealed that these compounds are well tolerated and are not cytotoxic to human subjects, but at the same time, they have limited therapeutic potential due to their high lipophilicity, rapid metabolism, and poor bioavailability [50]. This might be overcome by their specific usage in the form of gastroresistant tablets to treat inflammatory bowel disease patients or in the form of creams to treat psoriatic skin changes, as shown by Tian et al. [52] for oleanolic acid in a mouse model or by the use of nanoparticles as carriers [60].

## 4. Materials and Methods

### 4.1. Molecular Similarity

The existing library of 608 compounds (L1600 Kinase Inhibitor Library, TargetMol) was investigated to select promising candidates for biologically active ligands. To achieve this goal, the library was confronted with 11 ligands for which there is experimental evidence of such biological activity in the context of the RORγT nuclear receptor. For each compound contained in the library, as well as for the 11 species mentioned above, the topological fingerprints were calculated, as implemented in the RDKit library [61]. The molecular fingerprint is an abstract representation of certain structural features of the molecule [62]. The particular algorithm of topological fingerprint identifies all subgraphs in the molecule within a particular range of sizes, hashes each subgraph to generate a raw bit ID, and ultimately sets the corresponding bit within the resulting fingerprint. The default RDKit parameterization was applied, i.e., the range of subgraph sizes was 1–7, and the length of the resulting fingerprint was set to 2048. To find the similarities between the compounds from the library and the 11 RORγ ligands, the Tanimoto coefficients were calculated between relevant fingerprints. The Tanimoto coefficient is a simple function of two binary fingerprints and reflects the similarity extent of fingerprints [62,63]. The resulting lists of Tanimoto coefficients were sorted in descending order and directly provided the most similar species to each of 11 ROR-γ ligands.

### 4.2. Creation SMILES Codes, Stereoisomers, and Docking Simulations

In the current study, an exhaustive search of a complete space of stereoisomers was carried out. This effort was decomposed into the following steps: (1) creation of the SMILES codes for all stereoisomers, (2) turning the SMILES codes into the 3D structure, and (3) docking the 3D structures into the chosen receptor. To generate the SMILES code reflecting all the stereoisomers, we used the Gypsum-DL library [64]. The resulting SMILES representation was later turned into 3D structures with an in-house Python script based on the RDKit and OpenEye libraries [61,65,66]. Essentially, for each stereoisomer, explicit hydrogens were added followed by conformer generation. The size of the conformer population was limited to 10, and the geometry of each created conformer was relaxed with the Merck molecular force field (MMFF) [67]. The final structure of a particular stereoisomer was chosen according to the MMFF energy, i.e., the conformer with the lowest possible MMFF energy was taken for further treatment.

For the docking studies, the smina program was used, which is a fork of AutoDock Vina [68] coupled with the pyscreener library [69]. As a receptor, the crystallographic structure retrieved from the PDB database was chosen [70]; in particular, the 3l0j structure with a bound natural ligand—25-hydroxycholesterol—was taken [32]. To use this structure, a standard protocol transforming the experimental PDB structure into a form that is suitable for molecular docking was applied. In particular, the natural ligand and the solvent molecules were removed, the alternate conformations of the residues were properly handled, and the entire structure was checked against missing or incomplete residues. For all these preparatory efforts, the Chimera [71] and PMV [72] programs were used. Obtained in this way, the clean protein structure was later protonated within the PDB2PQR [73] software in order to obtain the protonation state relevant to pH = 7; herein, the PROPKA [74,75] method was applied with the AMBER force field. The protonated protein was subsequently converted into the pdbqt format, which is suitable for docking calculations. According to the docking protocol, both in the case of ligands and the receptor, the nonpolar hydrogens were merged, and the Gasteiger charges were calculated. Here, we used the ADFR software suite [72]. For the visualization of protein–ligand interactions the PyMOL software was used [76].

All considered stereoisomers were docked into the receptor within the Pyscreener module with the smina docking program in the backend. Pyscreener allows for the automation of the docking procedure and the application of high-throughput virtual screening within the Python ecosystem [69]. The docking calculation was oriented on the orthosteric pocket of the 3l0j receptor. The optimal pose search space was determined by a cube with an edge length of 25 Å centered in the geometrical center of the 3l0j native ligand.

### 4.3. Reagents

Ursolic acid, oleanolic acid, asiatic acid, and maslinic acid were purchased from Millipore Sigma (Burlington, MA, USA). Corosolic acid was purchased from Cayman Chemical Company (Ann Arbor, MI, USA).

### 4.4. Cell Viability

The cytotoxicity of ursolic, corosolic, oleanolic, asiatic, and maslinic acids in Th17 cells was determined using the CellTiter-Glo^®^ Luminescent Cell Viability Assay (Promega Cooperation, Fitchburg, WI, USA) according to the manufacturer’s protocol. CD4+ cells isolated from healthy donors were subjected to Th17 cell differentiation in the presence of increasing concentrations of the indicated compounds for 5 days. After that time, the luminescence of each sample was determined with an Infinite^®^ 200 PRO (Tecan Group, Männedorf, Switzerland).

### 4.5. Transfection and Luciferase Assay

The reporter vector pGL4.35[luc2P/9XGAL4UAS/Hygro] was purchased from Promega Cooperation (Madison, WI, USA). The GAL4-DBD RORγ fusion construct was described previously [35] and was a kind gift from Prof. Patrick Griffin. HEK293 cells were seeded into 96-well white plates at a density of 1 × 104 cells per well. The next day, they were cotransfected with pGL4.35[luc2P/9XGAL4UAS/Hygro], GAL4-DBD RORγ, and pCMV-SEAP (a kind gift from Dr. S. Schlatter, Zurich) vectors with TurboFect (Thermo Fisher Scientific, Waltham, MA, USA). After 24 h, cells were treated with increasing concentrations of the indicated compounds for the next 48 h. Following incubation, the cells were harvested and lysed, and the luciferase activity was determined using an Infinite^®^ 200 PRO (Tecan Group) with D-Luciferin (luciferase substrate) (Cayman Chemical Company). Alkaline phosphatase activity was determined spectrophotometrically at 405 nm in culture medium as a control of transfection efficiency.

### 4.6. Th17 Cells Differentiation

The naive CD4+ fraction was isolated using CD4 M-pluriBeads^®^ anti-hu (pluriSelect Life Science, Leipzig, Germany) from the buffy coats obtained from healthy, anonymous donors (buffy coats were purchased from the Regional Center for Blood Donation and Blood Treatment, Lodz, Poland). To differentiate naive CD4+ cells into human Th17 lymphocytes, the protocol by Wilson et al. [77] was used: cells were cultured in RPMI 1640 medium (PAN-Biotech, Aidenbach, Germany) containing 1% human AB serum and were treated with the following cytokines from PeproTech (Rocky Hill, NJ, USA): 50 ng/mL human IL-1b, 30 ng/mL human IL-6, 10 ng/mL human IL-23, 10 ng/mL human TGF-β, and beads coated with anti-CD2, anti-CD3, and anti-CD28 (T cell activation/expansion kit from Miltenyi Biotec, Bergisch Gladbach, Germany) for 5 days.

### 4.7. Real-Time RT–PCR

Total RNA from Th17 cells was isolated using TRI Reagent (Sigma-Aldrich, St. Louis, MO, USA). The reverse transcription of total RNA for cDNA synthesis was performed with a Maxima First Strand cDNA Synthesis Kit for RT-quantitative PCR (Thermo Fisher Scientific, Waltham, MA, USA). Real-time RT–PCR was conducted on a LightCycler 480 from Roche (Basel, Switzerland) using SYBR Green I Master Mix. The reaction conditions were as follows: 95 °C for 5 min, followed by 40 cycles of 95 °C for 10 s, 60 °C for 10 s, and 72 °C for 20 s. The following primer pairs were used: *RORγT*: 5′-CTGCTGAGAAGGACAGGGAG-3′ (forward) and 5′-AGTTCTGCTGACGGGTGC-3′; *IL-17A*, 5′-AAACAACGATGACTCCTGGG-3′ (forward) and 5′-CTTGTCCTCAGAATTTGGGC-3′ (reverse) described previously [17]; *IL-17F*, 5′-CTTTCTGAGTGAGGCGGC-3′ (forward) and 5′-TGGGAACGGAATTCATGG-3′ (reverse) described previously [78]; *IL-21*, 5′-TCCCAAGGTCAAGATCGC-3′ (forward) and 5′-CCCTGCATTTGTGGAAGG-3′ (reverse) described previously [79]; *IL22*, 5′-TGGCTGATAACAACACAGACG-3′ (forward) and 5′-GCTTTTGCACATTCCTCTGG-3′ (reverse), *APOD*, 5′-CCTTTGAGAATGGACGCTGC-3′ (forward) and 5′-AGTTCTCATAGTCGGTGGCC-3′ (reverse) described previously [80]. The mRNA levels were normalized by geometric means from 3 housekeeping genes: *HPRT1*, 5′-TGACACTGGCAAAACAATGCA-3′ (forward) and 5′-GGTCCTTTTCACCAGCAAGCT-3′ (reverse); *HMBS*, 5′-GGCAATGCGGCTGCAA-3′ (forward) and 5′-GGGTACCCACGCGAATCAC-3′ (reverse); *RPL13A*, 5′-CCTGGAGGAGAAGAGGAAAGAGA-3′ (forward) and 5′-TTGAGGACCTCTGTGTATTTGTCAA-3′ (reverse), as described by Vandensompele et al. [81].

### 4.8. Chromatin Immunoprecipitation (ChIP)

To perform chromatin immunoprecipitation, naive CD4+ cells were cultured in Th17-polarizing conditions for 5 days in the presence of ursolic acid, corosolic acid, and oleanolic acid. After that time, the cells were fixed with formaldehyde to cross-link proteins with DNA and then harvested and lysed. The DNA was subjected to sonication with a VCX-130 sonicator (Sonics & Materials Inc. (Newtown, CT, USA). An EZ-Magna ChIP A/G kit from EMD Millipore (Billerica, MA, USA) was used to immunoprecipitate the samples with the following antibodies: normal mouse IgG (EMD Millipore) and anti-ROR gamma antibody [162C2a] ab58670 from Abcam (Cambridge, UK). The relative enrichment of IL17A and IL17F promoters was analyzed with real-time PCR methodology. The reactions were performed under the following conditions: 95 °C for 10 min, 40 cycles of 95 °C for 20 s, 58 °C for 20 s, and 72 °C for 20 s. Primers complementary to *IL17A* and *IL17F* were described previously: [29] 5′-GCAGCTCTGCTCAGCTTCTA-3′ (forward, *IL17A*) and 5′-GGGCTTTTCTCCTTCTGTGG-3′ (reverse, *IL17A*); 5′-CTCTGATTTGTGGGCAATGG-3′ (forward, *IL17F*) and 5′-CCGGAGTTACTGACGAATGC-3′ (reverse, *IL17F*). The abundance of a specific promoter sequence was calculated using the dCt method with the Ct obtained for input DNA as a reference value as follows: 1000*2-dCt, where dCt = Ct sample Ct input DNA as described previously [26].

### 4.9. IL-17 ELISA

The concentrations of IL17 in cellular supernatants from naive CD4+ cells cultured under Th17-polarizing conditions in the presence of increasing concentrations of the indicated compounds were analyzed by ELISA using the Quantikine Human IL-17 Immunoassay kit (R&D Systems). The absorbance of the samples at 405 nm was read in a Sunrise microplate reader (Tecan).

### 4.10. Statistical Analysis

Statistical analysis was performed using Friedman repeated-measures ANOVA on ranks followed by Student–Newman–Keuls post hoc test. A *p* value of 0.05 or lower was considered statistically significant.

## 5. Conclusions

In summary, using the 2D fingerprint similarity search (Tanimoto coefficient ranges) we identified novel RORγT inverse agonists. Unfortunately, the calculation of free energy for the docked complexes of the RORγ-LBD did not allow us to distinguish the molecules with a high affinity to the ligand-binding domain (corosolic and oleanolic median values—10.85 kcal/mol and −11.3 kcal/mol, respectively) from those with a lower affinity (asiatic and maslinic acids median values: −10.4 kcal/mol and −11.3 kcal/mol, respectively), and experimental verification was needed. The presented methodology can be used to support experimental library screening, which, due to its specificity (e.g., selection of a nonoptimal concentration of the compounds for the screen), is at risk of losing a significant amount of information in the form of false-negative results.

## Figures and Tables

**Table 1:** The space of stereoisomers for each considered compound.

Compound.	Number of Chiral Centers	Number of Stereoisomers
Ursolic acid	10	1024
Corosolic acid	11	2048
Oleanolic acid	8	256
Maslinic acid	9	512
Asiatic acid	12	4096

**Table 2:** The collected minimum, maximum, and median values of the binding energies for all considered acids.

Compound	Minimum	Maximum	Median
		[kcal/mol]	
Ursolic acid	−13.7	−8.6	−11.0
Corosolic acid	−13.3	−6.6	−10.85
Oleanolic acid	−13.3	−7.5	−11.3
Maslinic acid	−13.6	−6.9	−11.3
Asiatic acid	−13.4	−6.2	−10.4

## Data Availability

All data for this study are included in the manuscript and the supplementary files.

## Supplementary Materials

The following supporting information can be downloaded at: https://www.mdpi.com/article/10.3390/ijms23031906/s1.
