# Supplementary material for: Identification of Corosolic and Oleanolic Acids as Molecules Antagonizing the Human RORγT Nuclear Receptor Using the Calculated Fingerprints of the Molecular Similarity
Source: Int J Mol Sci. 2022 Feb 8;23(3):1906. doi: 10.3390/ijms23031906 (PMC8837092; doi:10.3390/ijms23031906)
Supplement: Supplementary file 1 [file ijms-23-01906-s001.zip › Supplementary information.pdf]

Supporting information

Identification of the corosolic and oleanolic acids as molecules antagonizing the human RORγT nuclear receptor using the calculated fingerprints of the molecular similarity.

Joanna Pastwińska, Kaja Karaś, Anna Sałkowska, Iwona Karwaciak, Katarzyna Chałaśkiewicz, Błażej A. Wojtczak, Rafał A. Bachorz, and Marcin Ratajewski

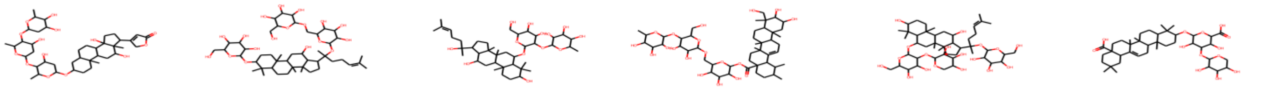

digoxin	0.657, Gypenoside XVII	0.654, Ginsenoside Rg2	0.653, Asiaticoside	0.648, Notoginsenoside R1	0.604, Momordin Ic

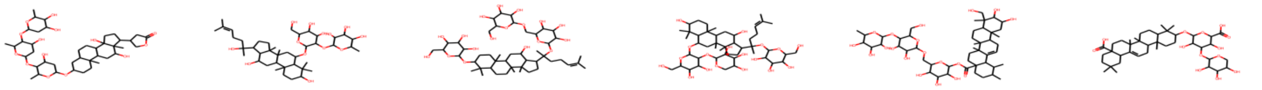

20,22-Dihydrodigoxin	0.721, Ginsenoside Rg2	0.712, Gypenoside XVII	0.703, Notoginsenoside R1	0.603, Asiaticoside	0.541, Momordin Ic

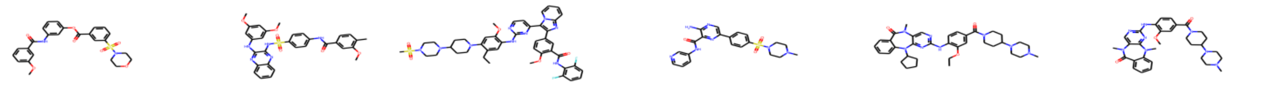

Oprea1_343674	0.487, SAR245409 (XL765)	0.45, GSK1904529A	0.439, AZD2858	0.432, XMD17-109	0.427, LRRK2-IN-1

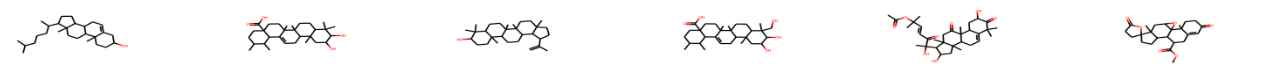

cholesterol	0.556, Corosolic acid	0.546, Lupeol	0.529, Asiatic acid	0.404, Cucurbitacin B	0.384, Eplerenone

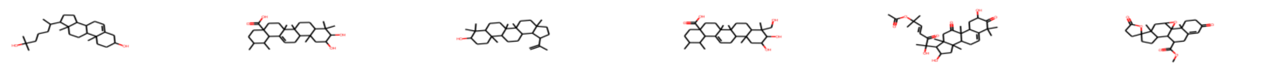

25-Hydroxycholesterol	0.552, Corosolic acid	0.536, Lupeol	0.525, Asiatic acid	0.413, Cucurbitacin B	0.392, Eplerenone

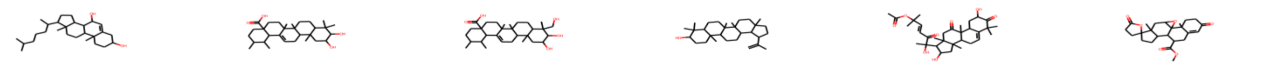

7alpha-Hydroxycholesterol	0.58, Corosolic acid	0.556, Asiatic acid	0.556, Lupeol	0.452, Cucurbitacin B	0.425, Eplerenone

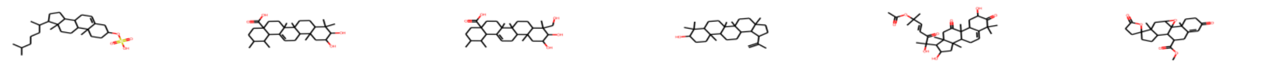

Cholesterol sulfate	0.485, Corosolic acid	0.472, Asiatic acid	0.413, Lupeol	0.397, Cucurbitacin B	0.392, Eplerenone

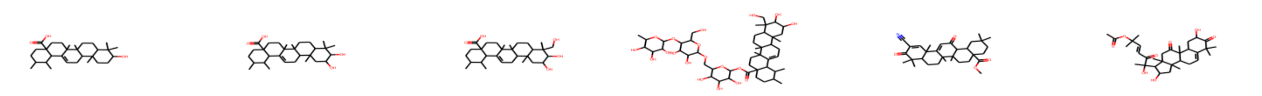

Ursolic acid	0.945, Corosolic acid	0.894, Asiatic acid	0.525, Asiaticoside	0.516, Bardoxolone Methyl	0.516, Cucurbitacin B

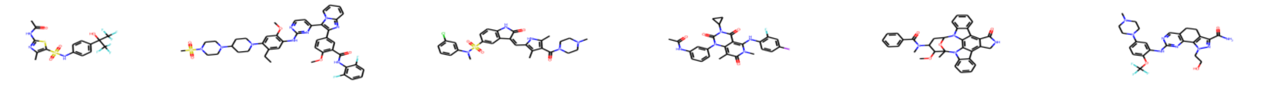

SR1001	0.452, GSK1904529A	0.451, SU11274	0.446, Trametinib (GSK1120212)	0.445, PKC-412(Midostaurin)	0.444, NMS-P937 (NMS1286937)

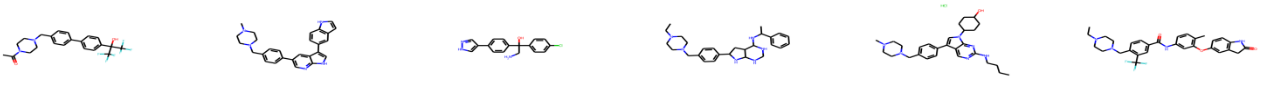

SR1555	0.368, URMC-099	0.342, AT13148	0.326, AEE788	0.322, UNC-2025	0.321, Ddr1-In-1

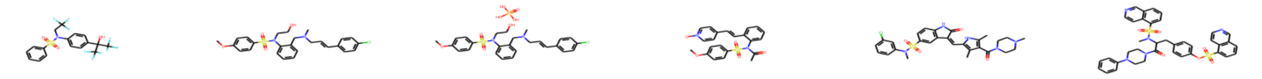

T0901317	0.544, KN-93	0.542, KN-93 Phosphate	0.524, HMN214	0.429, SU11274	0.405, KN-62

Figure S1. Results of the initial virtual screening of the L1600 Kinase Inhibitor Library (TargetMol).

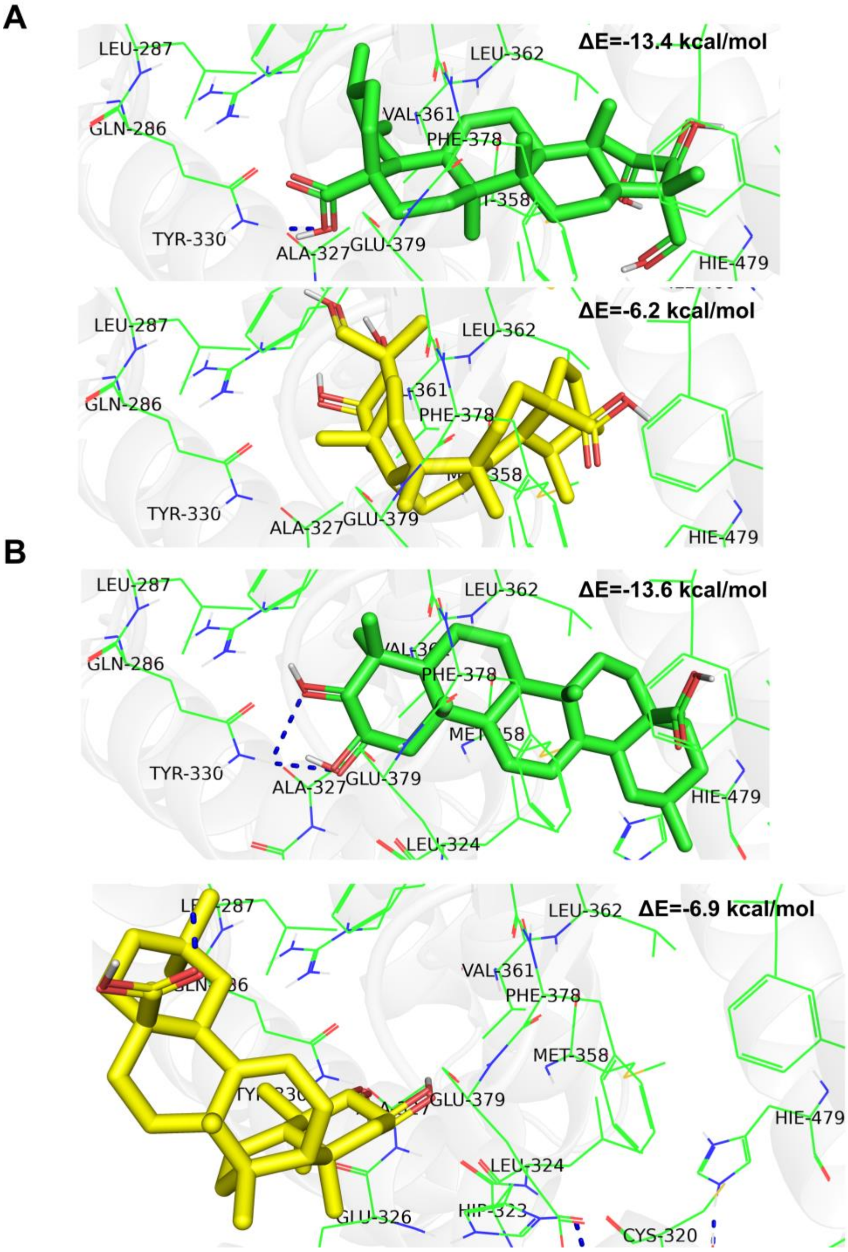

Figure S2. Molecular docking analysis of the best (green) and the worst (yellow) stereoizomers for asiatic (A), and malinic (B), acids binding to the LBD of the RORγ receptor. Hydrogen bonds are represented as dark blue dotted lines.

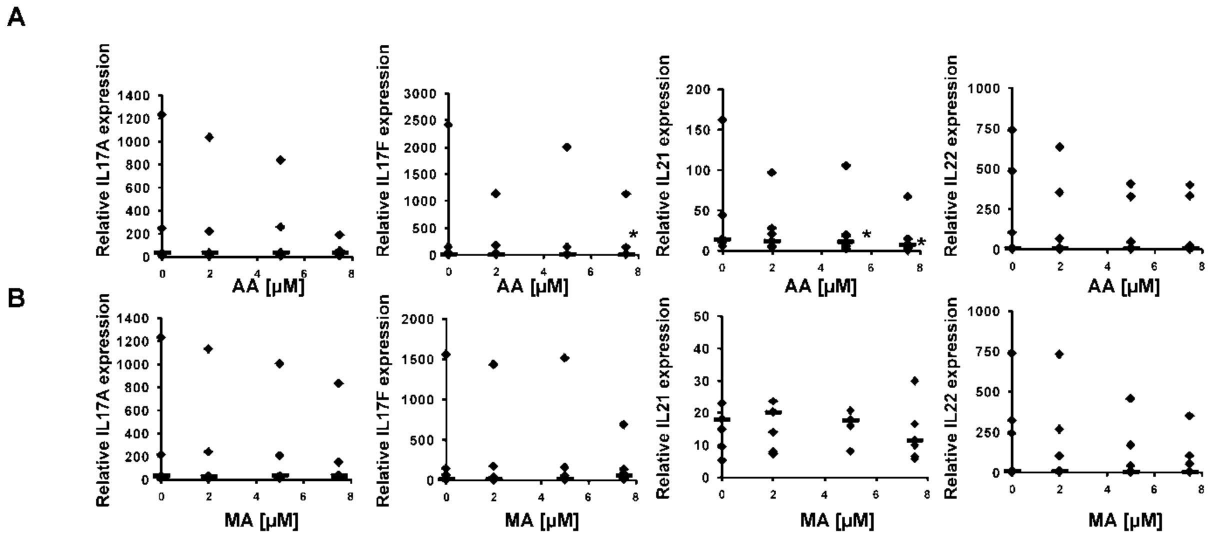

Figure S3. Effect of asiatic (A) and maslinic (B) acids on the expression of selected genes in human Th17 cells. Human naive CD4+ cells were treated with increasing concentrations of asiatic and maslinic acid acids and cultured under Th17 polarizing conditions for 5 days. Then, cells were collected for RNA extraction. The expression of the *IL17A*, *IL17F*, *IL21*, and *IL22* genes was determined by real-time RT–PCR. The results were normalized to the housekeeping genes *HPRT1*, *HMBS*, and *RPL13A*. An asterisk indicates a statistically significant difference at $p < 0.05$ compared with control cells. The data are presented as statistical dot plots with the median value (bars) from seven independent cultures (n = 7).

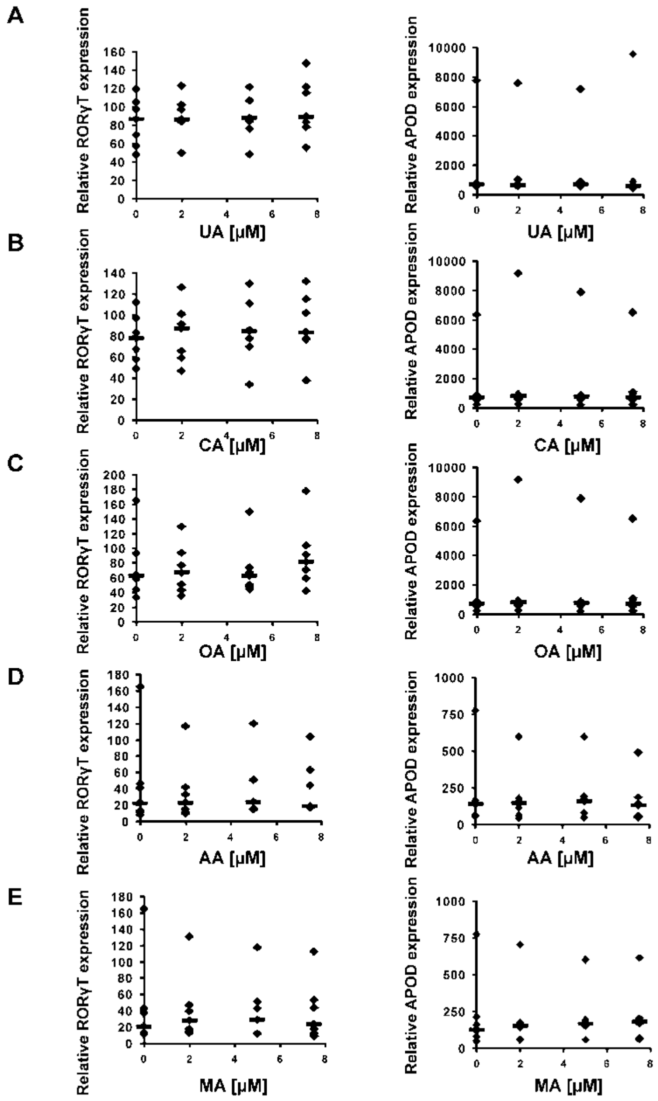

Figure S4. Ursolic acid analogs do not alter the expression of the *RORγT* and *APOD* genes in human Th17 cells. Human naive CD4+ cells were treated with increasing concentrations of ursolic (A), corosolic (B), oleanolic (C), asiatic (D), and maslinic (E) acids and cultured under Th17 polarizing conditions for 5 days. Then, cells were collected for RNA extraction. The expression of the *RORγT* and *APOD* genes was determined by real-time RT–PCR. The results were normalized to the housekeeping genes *HPRT1*, *HMBS*, and *RPL13A*. The data are presented as statistical dot plots with the median value (bars) from seven independent cultures (n = 7).

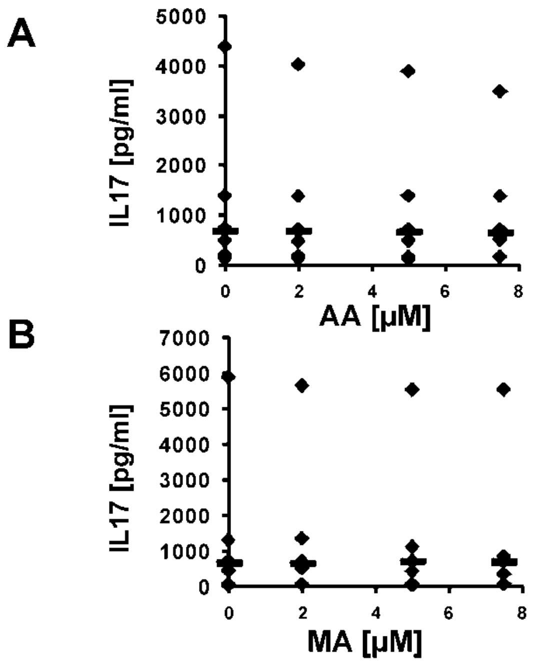

Figure S5. The analysis of IL-17 production in supernatants of Th17 cells cultured in the presence of increasing concentrations of asiatic (A) and maslinic (B) acids for 5 days was determined using the Quantikine Human IL-17 Immunoassay kit (R&D Systems). The data are presented as statistical dot plots with the median value (bars) from seven independent cultures (n = 7).
